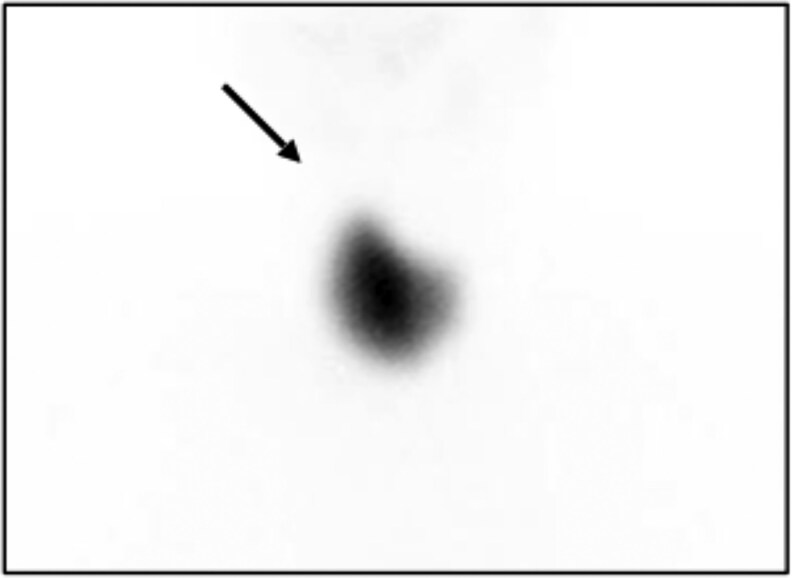# Thyroid lobe agenesis: a rare clinical finding

**DOI:** 10.1210/jcemcr/luag056

**Published:** 2026-04-29

**Authors:** Ivan Diaz-Cuadrado, Melissa Arias, Juan Carlos Baanante, Claudia Mitru

**Affiliations:** Department of General and Digestive Surgery, Hospital Universitari MútuaTerrassa, 08915 Terrassa, Barcelona, Spain; Department of General and Digestive Surgery, Hospital Universitari MútuaTerrassa, 08915 Terrassa, Barcelona, Spain; Department of General and Digestive Surgery, Hospital Universitari MútuaTerrassa, 08915 Terrassa, Barcelona, Spain; Department of General and Digestive Surgery, Hospital Universitari MútuaTerrassa, 08915 Terrassa, Barcelona, Spain

**Keywords:** thyroid, agenesis, congenital, scintigraphy, hyperthyroidism

## Image legend

A 28-year-old woman with primary hyperthyroidism due to Graves disease, diagnosed in July 2024, presented with hyperthyroidism refractory to treatment with propranolol and methimazole. Thyroid-stimulating immunoglobulins were markedly elevated at 22.51 IU/L (reference range, <0.55 IU/L).

The case was discussed at a multidisciplinary endocrinology board, and a thyroid ultrasound was performed as the initial imaging study. Ultrasound demonstrated diffuse enlargement of the right thyroid lobe with heterogeneous echotexture and patchy pseudonodular areas, suggestive of thyroiditis, along with a diffusely increased Doppler signal. Absence of the left thyroid lobe was noted, consistent with left thyroid hemiagenesis.

Subsequently, thyroid scintigraphy using technetium-99m pertechnetate (Tc-99m) was performed, showing a radiotracer uptake of 7.8% and confirming agenesis of the left thyroid lobe.

Thyroid lobe agenesis is a very rare congenital anomaly, with an estimated prevalence of 0.05% to 0.2%. It occurs more frequently in women and affects the left lobe more often than the right (∼80%) [1 ]. The condition is usually asymptomatic and is most often diagnosed incidentally during imaging studies performed for other thyroid disorders [2]. The remaining thyroid lobe may develop any thyroid pathology, and its evaluation and management should follow standard clinical criteria.

**Figure:**